# Clinical Validation of the Impact of Branch Stent Extension on Hemodynamics in ISF-TEVAR Involving LSA Reconstruction

**DOI:** 10.3389/fcvm.2022.911934

**Published:** 2022-06-13

**Authors:** Jiateng Hu, Fengshi Li, Peng Qiu, Xiaoyu Wu, Hongji Pu, Zhen Zhao, Jinbao Qin, Guang Liu, Shanliang Jin, Xinwu Lu, Xiaobing Liu

**Affiliations:** ^1^Department of Vascular Surgery, Shanghai Ninth People's Hospital, Shanghai Jiao Tong University School of Medicine, Shanghai, China; ^2^Vascular Centre of Shanghai Jiao Tong University, Shanghai, China; ^3^Department of Anesthesiology, Shanghai Ninth People's Hospital, Shanghai Jiao Tong University School of Medicine, Shanghai, China

**Keywords:** aortic dissection, endovascular treatment, left subclavian artery reconstruction, hemodynamics, branch stent extension length

## Abstract

**Background:**

The study of hemodynamics regarding thoracic endovascular aortic repair (TEVAR) is helpful to improve the surgical efficacy.

**Objective:**

Correlations between hemodynamic changes and branch stent extension length and interference factors for branch stent extension length of *in situ* fenestration TEVAR (ISF-TEVAR) involving the left subclavian artery (LSA) were evaluated.

**Materials and Methods:**

This study retrospectively analyzed 196 patients with Stanford type B aortic dissection who received *in situ* laser fenestrated thoracic endovascular aortic repair with LSA fenestration from April 2014 to March 2021. Branch stent extension to the main stent graft was evaluated by the computed tomographic angiography (CTA). Hemodynamic change of LSA was defined as a 20 mmHg interbrachial systolic pressure difference. The factors affecting the extension of the branch stent were also evaluated.

**Results:**

All patients underwent ISF-TEVAR with LSA fenestration, and there was no recurrence during the follow-up. The mean length of the branch stent extension was 10.37 ± 0.34 mm, which was used to divide the patients into long and short groups. Asymptomatic hemodynamic changes (defined as a 20 mmHg interbrachial systolic pressure difference) in LSA were observed in 61 patients undergoing ISF-TEVAR involving LSA fenestration. The Spearman correlation analysis showed extension length of a branch stent >1.5 cm elevated the risk of hemodynamic changes.

**Conclusion:**

Overall, we conclude that branch stent extension length >1.5 cm induced LSA hemodynamic changes. Appropriate shortening of the stent extension length can improve the curative effect of ISF-TEVAR, especially when faced with a type II/III aortic arch and stent angles of <30 degrees.

## Introduction

Aortic dissection involving the arch of the aorta represents a serious condition characterized by high mortality and sudden death for the patients (1, 2). To re-expand the true lumen and to treat visceral mal-perfusion in aortic dissection, thoracic endovascular aortic repair (TEVAR) was developed (3). However, the conventional TEVAR is not appropriate for aortic dissection involving the left subclavian artery (LSA). If the lesion is associated with a short proximal landing zone or has a primary entry tear at the aortic arch, TEVAR with LSA fenestration and the chimney technique are the alternative methods (4). To cope with the challenges related to complex morphological characteristics, *in situ* fenestration TEVAR (ISF-TEVAR) involving supplemental stents is proposed (5).

Theoretically, ISF-TEVAR can avoid changes in the anatomical structure or hemodynamics (6). A mismatch between the main endograft and the branch stent and insufficient support of the branch stent during TEVAR will cause symptoms of arterial hemodynamics disorders (7–9). In parallel stent graft technology, the hemodynamic disturbance of the branch artery is usually attributed to insufficient support of the branch stent (10). To our knowledge, in clinical practice of ISF-TEVAR involving LSA reconstruction, the modification mode of the main stent and the connection between the main stent and the branch stent are not completely consistent with theoretical research. For instance, the branch stent often protrudes into the aortic arch to prevent migration, which may lead to branch stent overextension (11, 12). Additionally, fenestration and branch stent implantation for type III aortic arches tends to trigger long-term complications caused by narrow angles or long-stent extension lengths. It has been confirmed *in vitro* that shortening the extension length of the stent graft may improve the efficacy of ISF-TEVAR from the perspective of hemodynamics (13). While the previous studies have evaluated the effects of *in situ* LSA fenestration on hemodynamics, there is a lack of validation and exploration from the clinical perspective.

The ISF-TEVAR has been the primary approach for lesions involving the aortic arch in our center since 2014 (14). In this article, we are committed to exploring the relationship between stent extension length and hemodynamics in ISF-TEVAR requiring LSA fenestration in order to offer novel insights in guiding ISF-TEVAR operation. In addition, the further analysis was applied to confirm the critical factors that influence the length of branch stent extension based on the clinical baseline characteristics and computed tomographic angiography (CTA) follow-up information of Stanford type B aortic dissection patients.

## Materials and Methods

This study was approved by the Shanghai Ninth People's Hospital Ethics Committee, Shanghai Jiao Tong University School of Medicine. All patients signed informed consent for the operative procedures and data collection.

### Patient Identification

Between April 2014 and March 2021, a total of 252 patients were admitted to our center with Stanford type B aortic dissection (TBAD), among which 196 consecutive patients received ISF-TEVAR of the LSA. All patients met the inclusion criteria of the ISF-TEVAR procedure to reconstruct the LSA branches. Namely, only if the lesion involved the LSA or the proximal landing zones of the main stent were insufficient (defined as a distance ≤ 15 mm) would LSA fenestration be considered. Meanwhile, the exclusion criteria were as follows: (1) patients diagnosed with severe hepatic and renal insufficiency; (2) the proximal aortic healthy length between the LSA ostium and the proximal aortic hematoma > 1.5 cm; (3) a lesion involving the LCCA; and (4) incomplete or vague imaging follow-up data.

### Endovascular Procedures

**Figure 1:**
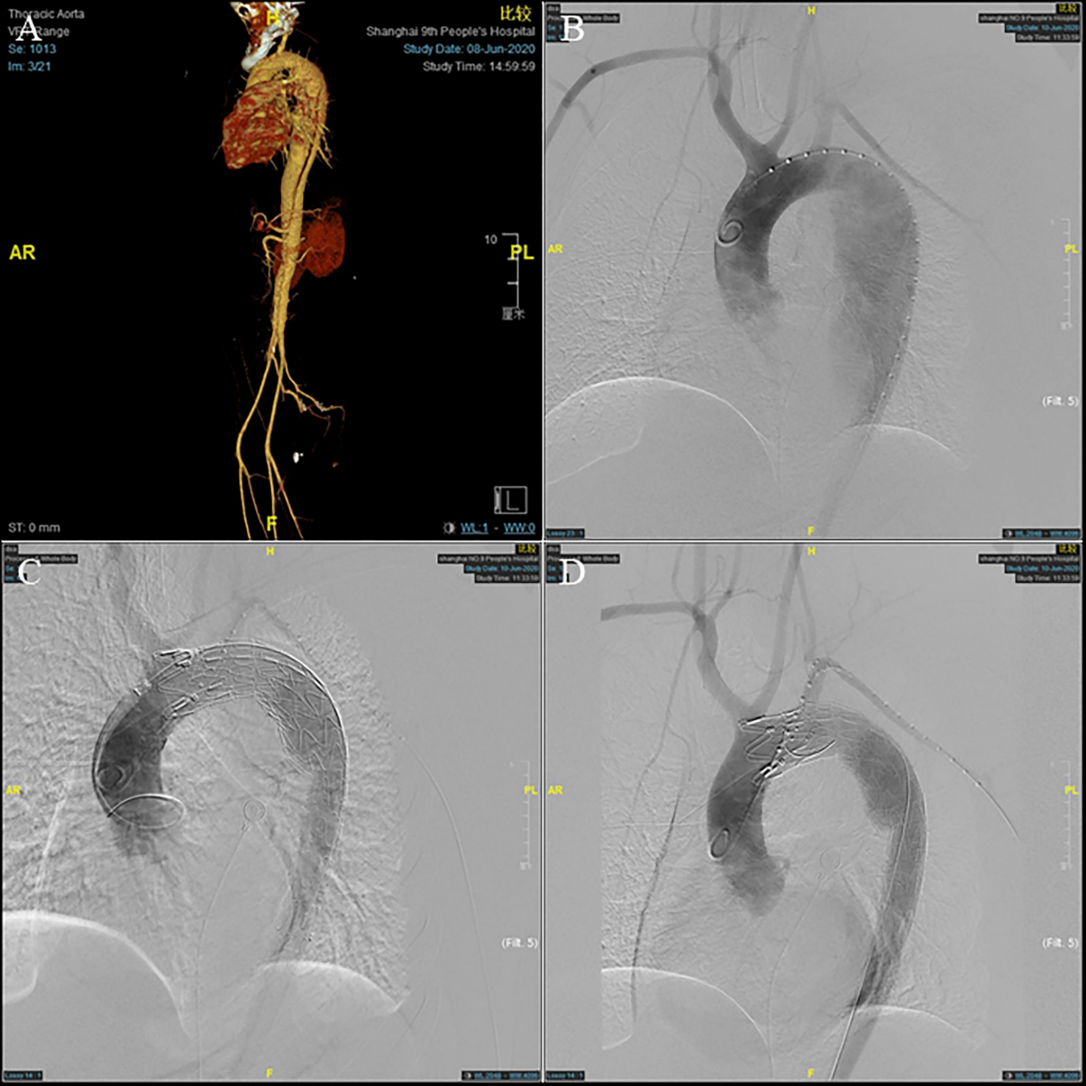
A 45-year-old man who presented with 7 days of chest and back pain was diagnosed as type B aortic dissection (TBAD). **(A)** Pre-operative computed tomographic angiography (CTA) and **(B)** intra-operative angiography showed a pathologically changed aorta arch. **(C)** The stent graft was deployed to seal the aortic arch lesion, leaving its distal end partially within the restrictive covered stent (RCS). **(D)** After left subclavian artery (LSA) fenestration, post-operative angiography showing satisfactory repair of the dissected aorta.

The preoperative evaluation was based on CTA, including the location of the primary entry tear and the diameter of the true lumen (Figure 1A). The detailed ISF-TEVAR procedure was described as follows: (1) local sterilization and anesthesia. (2) puncture of the femoral artery and the left brachial artery. The femoral artery and the left brachial artery were punctured or surgically exposed. Subsequently, 8F vascular sheaths (Terumo Corporation, Tokyo, Japan) were introduced through the femoral artery and the left brachial artery while intravenous heparin (5,000 U) was applied. An 8- or 6-F 55 cm sheath (Cook, Inc., Bloomington, Indiana) was inserted over a stiff wire to the origin of the LSA. Meanwhile, two 150 cm 4-F pigtail catheters (Cook, Inc.) over a 0.035-inch stiff guidewire (Terumo Corporation) were advanced into the ascending aorta, followed by angiography examination to confirm the length of the stent graft to be implanted. (3) Placement of the thoracic endograft. The thoracic endograft (GORE TAG; W.L. Gore & Associates, Inc., Flagstaff, Arizona; Valiant; Medtronic, Minneapolis, Minnesota; Ankura; Lifetech Scientific Co., Ltd., Shenzhen, China) was delivered through the femoral artery with oversizing <10% (5% on average) of the proximal aortic diameters between the LSA and the left common carotid artery (LCCA). Distal tapered stents (Ankura) were deployed in the aortic true lumen if necessary (Figures 1B,C). The details of the procedure are described in the Supplementary Video 1. (4) Laser fenestration of the LSA. An 810 nm ocular fiber (Eufoton S.R.L., Trieste, Italy) was assembled with a 4 mm × 40 mm balloon catheter (Mustang; Boston Scientific, Marlborough, Massachusetts), the distal end of which was exposed ~0.5 cm. In addition, the back end of the balloon catheter was connected to the Y valve to fix the optical fiber. We pushed the SG with a slight forward force. The angle of the long sheath was adjusted to make the ocular fiber head perpendicular to the internal graft as far as possible. The C arm X-ray machine was positioned in the right forward oblique position to determine the relative position of the ocular fiber and the stent graft. Subsequently, the stent graft was cauterized by a laser at a wavelength of 810 nm at 14–18 W and held for 3 s. (5) The ocular fiber was passed through the newly created fenestration. The balloon catheter was deployed into the lumen over the fiber. Then, appropriate expansion was performed on the balloon to predilate the round fenestration until the “gourd-shaped” stenosis disappeared. Finally, a covered stent 8–12 mm in width and 40 mm in length or a bare stent 8–10 mm in width and 39 mm in length was advanced into the lumen through the left subclavian artery. Then, an angioplasty balloon (diameter 8–12 mm) consistent with the fenestration stent diameter was typically utilized to stretch the stent section. The postoperative angiography demonstrated an accurate stent position, no incidence of endoleaks, satisfactory aortic branches, and LSA fenestration patency (Figure 1D).

### Follow-Up Protocol

Demographic data, clinical cardiovascular complications, aortic anatomical features, and operative data were collected. All patients underwent CTA at the first 3-month visit and annually thereafter. Outpatient or telephone follow-up was also performed within 1, 3, and 6 months of surgery. The primary clinical endpoint of this study was any branching stent-related reintervention, such as endoleak, stent rupture, displacement, ischemic symptoms of the left upper limb, and subclavian artery steal syndrome. The secondary clinical endpoint of the study was the hemodynamic change in LSA (defined as a 20 mmHg interbrachial systolic pressure difference) (15, 16). It is measured by taking the blood pressure in both arms after the patient sits still in a quiet clinic for at least 5 min, following standard procedures (17). The average value of the three measurements was taken.

### Data Collection and Definitions

**Figure 2:**
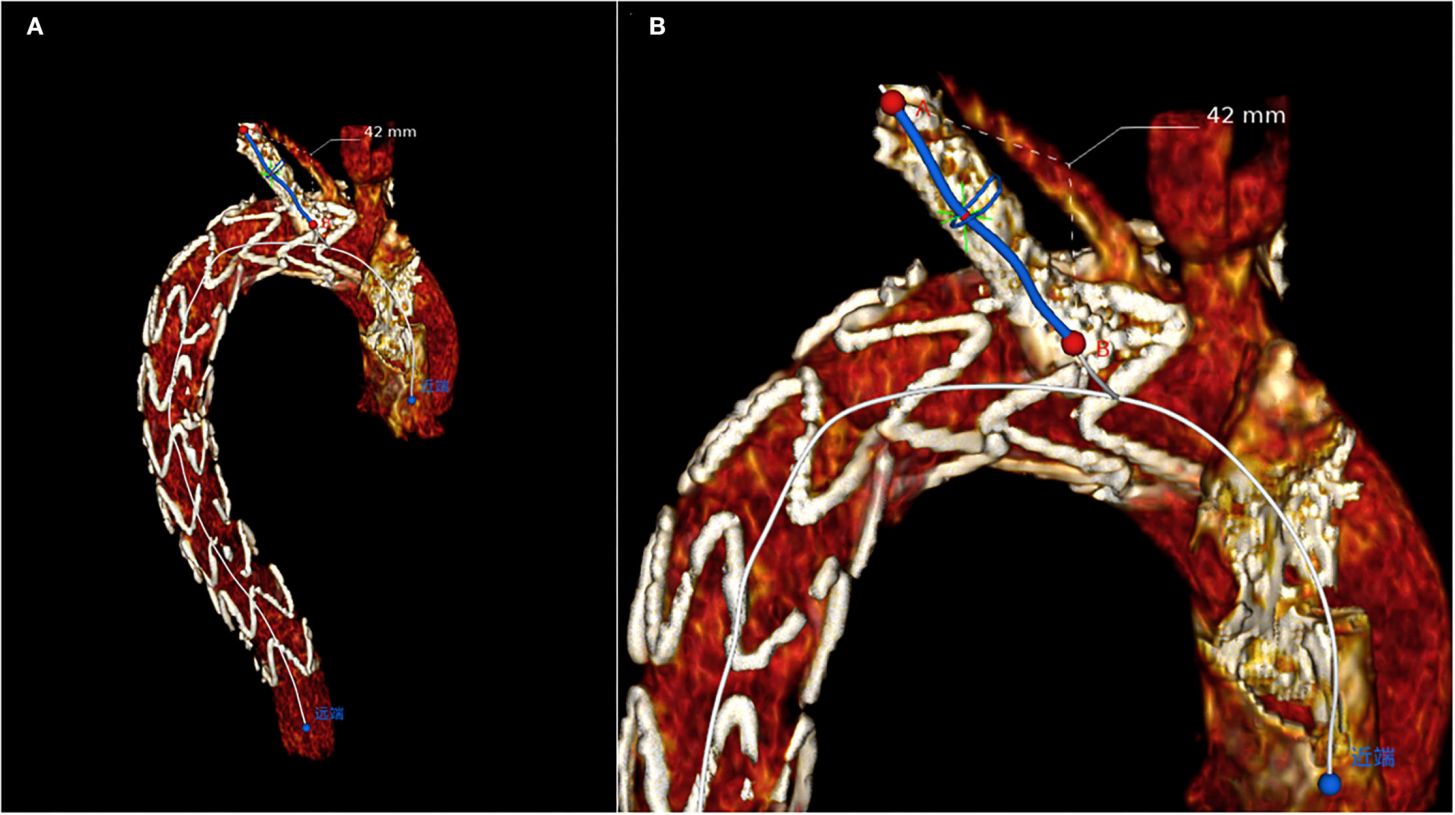
Plane of CTA image adjustment principle: Adjust the CTA image plane from all directions until the observer's line of sight is perpendicular to the LSA plane. **(A)** The overall view. **(B)** The magnified partial view.

**Figure 3:**
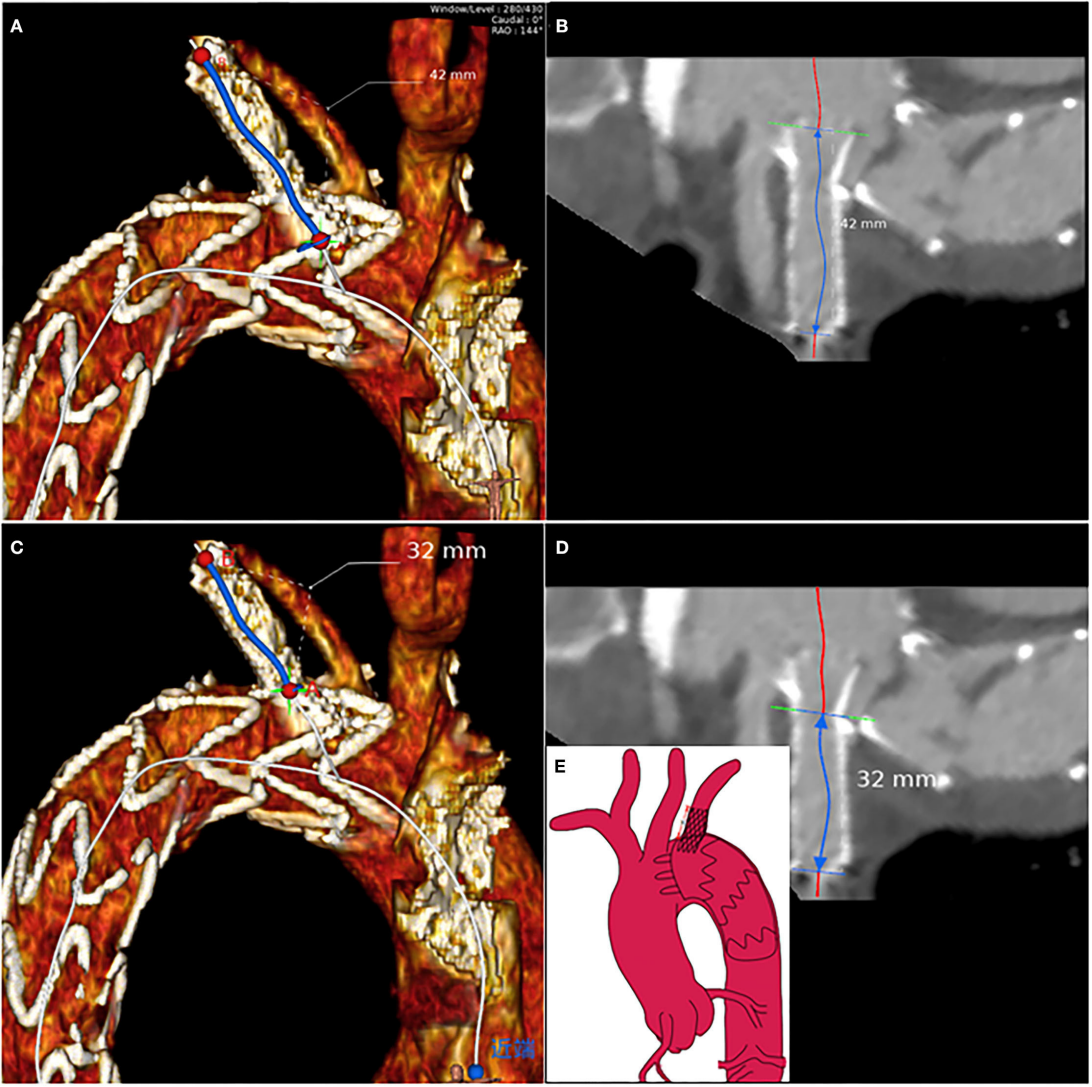
The branch stent protrusion length calculation protocol based on the multiplanar reconstruction: axial visualization of LSA to measure **(A)** the total length of branch stent and **(C)** the length of branch stent in LSA according to the center lines, respectively **(B,D)**. **(E)** The schematic suggested that the branch stent protrusion length was equal to the total branch stent length minus the length of branch stent in the LSA.

Morphological characteristics of the main and branch stents were collected from the latest follow-up CTA images. According to the Myla classification, the aortic arch types were classified into type I, type II, and type III. These reconstruction images were analyzed by using case planning software (EndoSize®, Therenva) designed for endovascular procedures based on the three-dimensional volume rendering and multiplanar reconstruction analysis protocols. The angle of the branch stent was defined by the angle of the tangent between the branch stent and the opening of the left subclavian artery, shown in the Supplementary Video 2. The fenestration position of the LSA branch stent can be divided into P0, P1, and P2 (P0: not affected by the metal structure of the main endograft; P1: affected by one metal structure; P2: affected by two metal structures) (Supplementary Figure S1). For example, postoperative three-dimensional (3D) computed tomography angiography reconstruction of these three different fenestration positions was shown in Supplementary Figure S2. The length of branch stent is defined as the length along the center line [i.e., the line that ideally coincided with the center of the arterial lumen (18)] (Figure 2), in which the observer's sight should be perpendicular to the plane where the branch stent was located. In detail, Supplementary Material presented the procedure of adjusting the view plane (Supplementary Video 3). Thereafter, the EndoSize® software automatically established the center line and performed the curved planar reconstruction, the axis of which was perpendicular to the center line. According to the curved planar reconstruction, the length of the branch stent could be calculated accurately (Figures 3A–D). Additionally, the extension length of branch stent was calculated by subtracting the length in the LSA from the overall length of branch stent, exhibited in Figure 3E. Then the patients were divided into long and short groups based on the average value of branch stent extension length. The entire measurement and grouping process was carried out by two experienced vascular surgeons.

### Statistical Analysis

The SPSS 21.0 software (IBM Corporation, Armonk, NY, USA) was used to perform the statistical analysis. The GraphPad Prism 8.0 (GraphPad Software, San Diego, USA) was utilized to create and plot the charts. The results for the continuous variables are reported as the mean ± standard deviation and number (%). The Fisher's exact test was performed for the classification variables. Interference factors of the branch stent extension length were evaluated by linear regression analysis. The Spearman correlation test was applied to analyze the relationship between branch stent extension length and hemodynamic changes. A *p*-value < 0.05 was considered to be statistically significant.

## Results

### Patient Characteristics and Intraoperative Details

**Figure 4:**
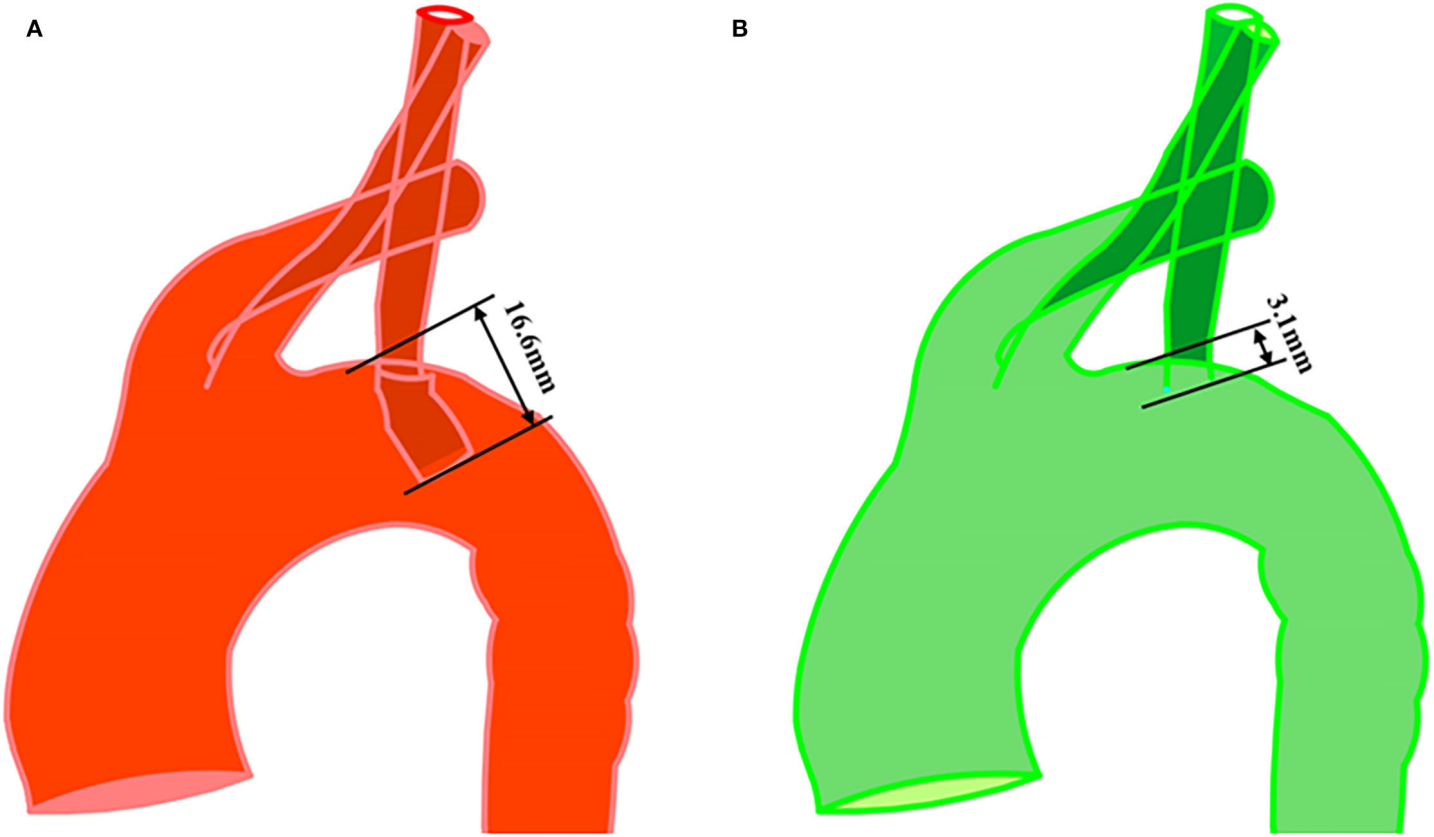
Measurement of branch stent protrusion length. **(A)** Maximum value (16.6 mm) and minimum value (3.1 mm) were shown in postoperative geometry with *in situ* fenestration. **(B)** The observer's sight was perpendicular to the plane where the branch stent was located.

The average age of the enrolled patients was 60.8 ± 8.4 years. One patient suffered from left arm fatigue at 10 months postoperatively, which was in the long extension length group. All patients underwent percutaneous endovascular angioplasty (PTA) and LSA fenestration, and there was no recurrence during follow-up. Detailed demographic and intraoperative characteristics of the patients are presented in Table 1. The mean length of the branch stent extension was 10.37 ± 3.44 mm. The maximum was 16.60 mm, while the minimum was 3.10 mm, as presented in Figure 4.

**Table 1:** Patient characteristics and baseline.

	**Number (%)/Mean ±Standard deviation**
Age-y	60.8 ± 8.4
Male	117 (59.7%)
**Cardiovascular complications**	
Hypertension	134 (68.4%)
Peripheral vascular diseases	50 (25.5%)
Coronary heart diseases	14 (7.1%)
Surgery time (min)	55.2 ± 11.2
Amount of contrast agent (ml)	98.6 ± 13.0
X-ray time (min)	39.8 ± 7.3
Branch stent extension length (mm)	10.37 ± 3.44
**Main body stents**	
TAG	77 (39.3%)
Valiant	68 (34.7%)
Ankura	51 (26.0%)

### Follow-Up Data

The mean follow-up was 26.08 ± 17.73 months (range, 3–57 months), and no stent rupture, displacement, or subclavian artery steal syndrome was observed during the process. Of the 196 total ISF-TEVAR involving LSA reconstruction cases, 98 were defined as a long extension length of the branch stent according to the mean length. Moreover, the types of the main body stents, branch stent angle, and aortic arch type are shown in Table 2.

**Table 2:** Follow-up computed tomographic angiography (CTA) data of the patients.

	**Number (%)**
**Fenestration location**	
P0/P1/P2	146/40/10
**Aortic arch**	
I/II/III	69/63/64
**Branch stent angle**	
0–30°/30–60°/60–90°	55/77/64
**Branch stent extension length (compared with mean)**	
Short/Long	98/98

*Mean = 10.37 ± 0.34 mm*.

### Correlation Between Hemodynamic Change and Extension Length of the Branch Stent

**Figure 5:**
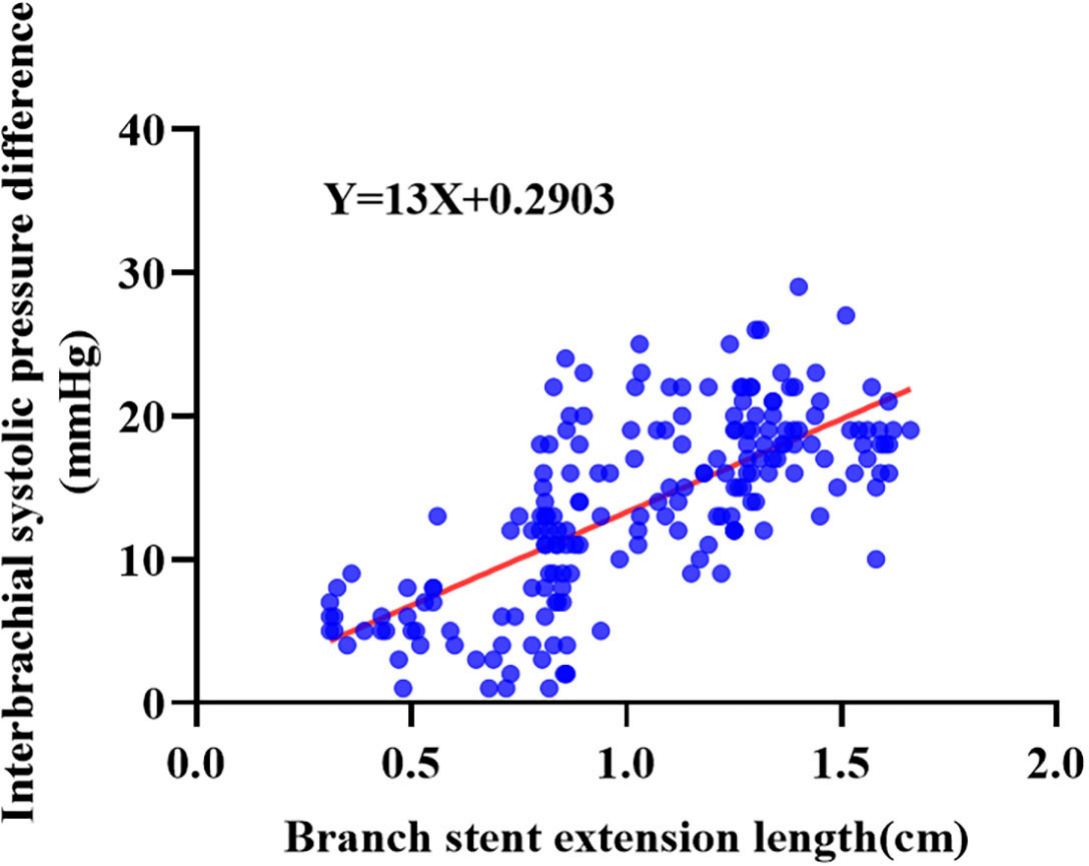
The Spearman correlation analysis of hemodynamic changes and branch stent extension length of the 196 enrolled patients receiving ISF-TEVAR including the LSA fenestration.

Asymptomatic hemodynamic changes in LSA were observed in 61 patients undergoing ISF-TEVAR involving LSA fenestration. The hemodynamic changes in the long group were more significant than that in the short group (Table 3). Spearman correlation analysis showed that the correlation coefficient was 0.493 (Table 3). Followed by function fitting, it was intuitively concluded that when the branch stent extension length was >1.516 cm, the risk of hemodynamic change (blood pressure change > 20 mmHg) was increased in Figure 5 (*p*-value < 0.001). The patients with asymptomatic hemodynamic changes were closely observed without further intervention.

**Table 3:** Correlation between hemodynamic changes and branch stent extension length.

	**Short (*n* = 98)**	**Long (*n* = 98)**
Hemodynamic change (+)	26	43
Hemodynamic change (-)	72	55

*Correlation coefficients: 0.493; P-value < 0.0001*.

### Factors Contributing to the Extension Length of the Branch Stent

Among the eight variables assessed as potential risk factors, no baseline variables affected the stent extension length. In terms of morphological variables, the aortic arch type and angle of the branch stents were significantly related to the stent extension length. Specifically, both type II (*T* value = 2.804, *p*-value < 0.01) and III aortic arches (*T*-value = 5.178, *p*-value < 0.001) were risk factors for the extension length of the branch stents. Furthermore, there was a significant relationship between the angle of the stent and the extension length of the stent. In detail, compared with the stent angle varying from 30 to 90 degrees, the group of 0–30 degrees (*T* value = 4.672, *p*-value < 0.001) was responsible for the extension of the fenestration stent length. The other factors had no significant correlation with the dependent variable (Table 4). Briefly, the results above indicated that the increase in the aortic arch curvature and the decrease in the LSA angle both increased the likelihood of branch stent extension.

**Table 4:** Linear regression analysis of interference factors for branch stent extension length.

**Variables**	**Regression coefficient**	**STD error**	***T*-value**	***P*-value**
Age-y	0.001	0.003	0.469	
Hypertension	−0.085	0.048	−1.766	0.708
Peripheral vascular diseases	0.010	0.051	0.187	0.885
Coronary heart diseases	0.084	0.088	0.953	0.333
**Stent location**				
P0	1.00	-	-	
P1	−0.041	0.103	−0.398	0.693
P2	0.069	0.051	0.713	0.608
**Main body stents**				
TAG	1.00	-	-	-
Valiant	−0.067	0.053	−1.266	0.205
Ankura	0.000	0.056	−0.004	0.984
**Branch stent angle**				
0–30°	0.285	0.061	4.672^***^	0.000
30–60°	−0.109	0.059	2.419^*^	0.016
60–90°	1	-	-	-
**Aortic arch type**				
I	1.00	-	-	-
II	0.155	0.055	2.804^**^	0.007
III	0.290	0.056	5.178^***^	0.000

* *P-value < 0.05*,

** *P-value < 0.01*,

*** *P-value < 0.001*.

## Discussion

Regarded as a promising technique for the revascularization of aortic branches, ISF-TEVAR is distinguished by a satisfactory 5-year survival rate and low postoperative complication rate (19, 20). Postoperative problems mainly include endograft migration or collapse, stent kinking and/or stenosis, and endograft infection following ISF-TEVAR. Our previous work demonstrated a satisfactory 5-year outcome of patients suffering from aortic dissection or aneurysm and receiving ISF-TEVAR (14). Consistently, no postoperative complications, such as stent rupture, occlusion, or LSA steal syndrome, occurred in the present study. When LSA reconstruction is required during ISF-TEVAR, a single fenestration will be created in the proximal portion of the main endograft. Meanwhile, the LSA branch stent protruding into the aortic arch can avoid endograft migration (21). Defined as displacement of the endograft by more than 5–10 mm from its original position, endograft migration has been reported to occur following 1.0–2.8% of TEVAR operations and 1–10% of endovascular repair procedures for the abdominal aorta at 1-year postintervention (22, 23). Thus, the patients in our center who underwent ISF-TEVAR did not experience stent displacement.

The sealing of the LSA has been demonstrated to be associated with stroke, arm ischemia, and spinal cord ischemia (SCI) (21, 24). By quantifying the impact of LSA sealing on hemodynamic parameters during TEVAR virtually, blood flow was investigated in a type B aortic dissection undergoing ISF-TEVAR with LSA reconstruction (25). The amount of blood flow crossing the LSA was between 3.52 and 5.28% in healthy young humans, which should vary from 4.01 to 6.14% under ideal conditions (26). However, the present outcome showed that merely 2.36% of the total inlet flow was assigned to the fenestration LSA, which was lower than expected. Furthermore, the configuration of the fenestration stent and the pressure on the stent surface were confirmed to promote stent contraction or migration (27). There is no clear standard for the protrusion length of the LSA fenestration stents, which mainly depends on the operator's experience. Interestingly, the previous research on hemodynamics according to computational fluid dynamics (CFD) revealed that branch stents have a strong influence on hemodynamics and that the protrusion length needs to be shortened properly after ISF-TEVAR (28, 29). As the protrusion length of the fenestration stent was shortened, a slight reduction in blood flow through the LCCA was observed. Moreover, the extension length of the fenestration stent also had an impact on the distribution of wall shear stress-related indices and the oscillatory nature of the flow (30). The upper and lower half of the branch stent had different effects on energy loss during a cardiac cycle (13). However, long-term follow-up results associated with hemodynamics remain scarce at present. With respect to the shortcomings of the existing studies mentioned above, a certain number of clinical subjects are required for further validation.

In the present study, we chiefly focused on the hemodynamic consequences of the differing protrusion lengths of the fenestration stent and evaluated the effects of the different interfering factors on stent extension length from a clinical perspective. The results of Spearman correlation analysis revealed that the extension length of branch stent >1.5 cm elevated the risk of hemodynamic changes, uncovering a novel strategy to guide the clinical work of ISF-TEVAR in the future. In addition, the CTA follow-up images revealed that 61 enrolled patients showed asymptomatic hemodynamic changes in LSA, which might be due to the fact that the morphologic changes occurred in short segments of the branch stents. Moreover, the protrusion zones of the supplemental device tend to be located at the opening of the LSA, a certain distance from the vertebral artery with abundant collateral circulation. Therefore, the stent extension length had little impact on the blood flow of the vertebral artery. Consistently, no cases of left vertebral artery occlusion were observed during postoperative follow-up. Nevertheless, hemodynamic changes are common in patients with stent protrusion. In addition, 20 mmHg has been identified as having an increased risk of long-term cardiovascular events (31). This group of patients was closely followed up after ISF-TEVAR. Moreover, branch stent angle and aortic arch type were significantly associated with stent protrusion length. Concretely, type II/III aortic arch and LSA angles <30 degrees are common in clinical work and proved to be pivotal factors of stent protrusion in this study. The curvature in the aortic arch of type III increases obviously. These anatomical conditions are often accompanied by distortion of the laser fiber approach, making it difficult to advance the coating structure of the mainframe, leading to an unsatisfactory fenestration shape. Hence, a more suitable device may be necessary for ISF-TEVAR as experimentation and knowledge advance.

### Study Limitations

There are some inherent limitations of this study. The first limitation is that our work is a retrospective single-center study. A larger sample size and long-term follow-up data are necessary to confirm the relationship between branch stent protrusion length and hemodynamic changes in ISF-TEVAR involving LSA reconstruction. Moreover, we deduced that aortic arch type and LSA angle could affect the stent extension length based on the follow-up data, but *in vitro* mechanical experiments are required to support this hypothesis. Finally, the factors that may affect the stent extension length included in our evaluation process are not sufficient theoretically, and additional factors are required for screening. Furthermore, some patients feel subjectively uncomfortable without showing definite hemodynamic changes.

## Conclusion

On the basis of the follow-up outcome of patients suffering from type B aortic dissection, the present study investigated the correlation between hemodynamic changes and the extension length of branch stents after ISF-TEVAR with LSA reconstruction. In addition, factors contributing to the extension length of the branch stent were also evaluated in conjunction with various analysis methods. Finally, we concluded that the branch stent protrusion length was related to LSA hemodynamic changes. The extension length of branch stent >1.5 cm elevated the risk of hemodynamic changes. Branch stent angle and aortic arch type were significantly associated with stent protrusion length. Appropriate shortening of the stent extension length can improve the curative effect of ISF-TEVAR, especially when faced with a type II/III aortic arch and stent angles of <30 degrees. Generally, our study demonstrated that shortening the branch stent protrusion length has the potential to improve the postoperative hemodynamic changes and aid vascular surgeons in achieving positive surgical results.

## Data Availability Statement

The raw data supporting the conclusions of this article will be made available by the authors, without undue reservation.

## Ethics Statement

The studies involving human participants were reviewed and approved by the Shanghai Ninth People's Hospital Ethics Committee, Shanghai Jiao Tong University School of Medicine. The patients/participants provided their written informed consent to participate in this study.

## Author Contributions

XLi, XLu, and SJ designed the study and analyzed the data. JH and FL drafted the article. PQ took on the task of CTA reconstruction. XW, HP, ZZ, JQ, and GL were responsible for the language correction. All authors finally approved the paper.

## Funding

The study was sponsored by the National Natural Science Foundation of China (81970405 and 82170488) and the Natural Science Foundation of Shanghai (21ZR1437300).

## Conflict of Interest

The authors declare that the research was conducted in the absence of any commercial or financial relationships that could be construed as a potential conflict of interest.

## Publisher's Note

All claims expressed in this article are solely those of the authors and do not necessarily represent those of their affiliated organizations, or those of the publisher, the editors and the reviewers. Any product that may be evaluated in this article, or claim that may be made by its manufacturer, is not guaranteed or endorsed by the publisher.

## Supplementary Material

Supplementary Figure S1 Schematic diagram of different fenestration positions of LSA branch stent: P0: not affected by the metal structure of the main endograft; P1: affected by one metal structure of the main endograft; P2: Affected by two metal structures of the main endograft.

Supplementary Figure S2 Postoperative three-dimensional (3D) computed tomography angiography reconstruction image showed different fenestration positions of LSA branch stents. **(A)** LSA branch stent was not affected by metal structure (P0). **(B)** LSA branch stent was affected by one metal structure (P1). **(C)** LSA branch stent was affected by two metal structures (P2).
