# Supplementary figures and images for: Clinical Validation of the Impact of Branch Stent Extension on Hemodynamics in ISF-TEVAR Involving LSA Reconstruction
Source: Front Cardiovasc Med. 2022 Jun 13;9:911934. doi: 10.3389/fcvm.2022.911934 (PMC9234204; doi:10.3389/fcvm.2022.911934)

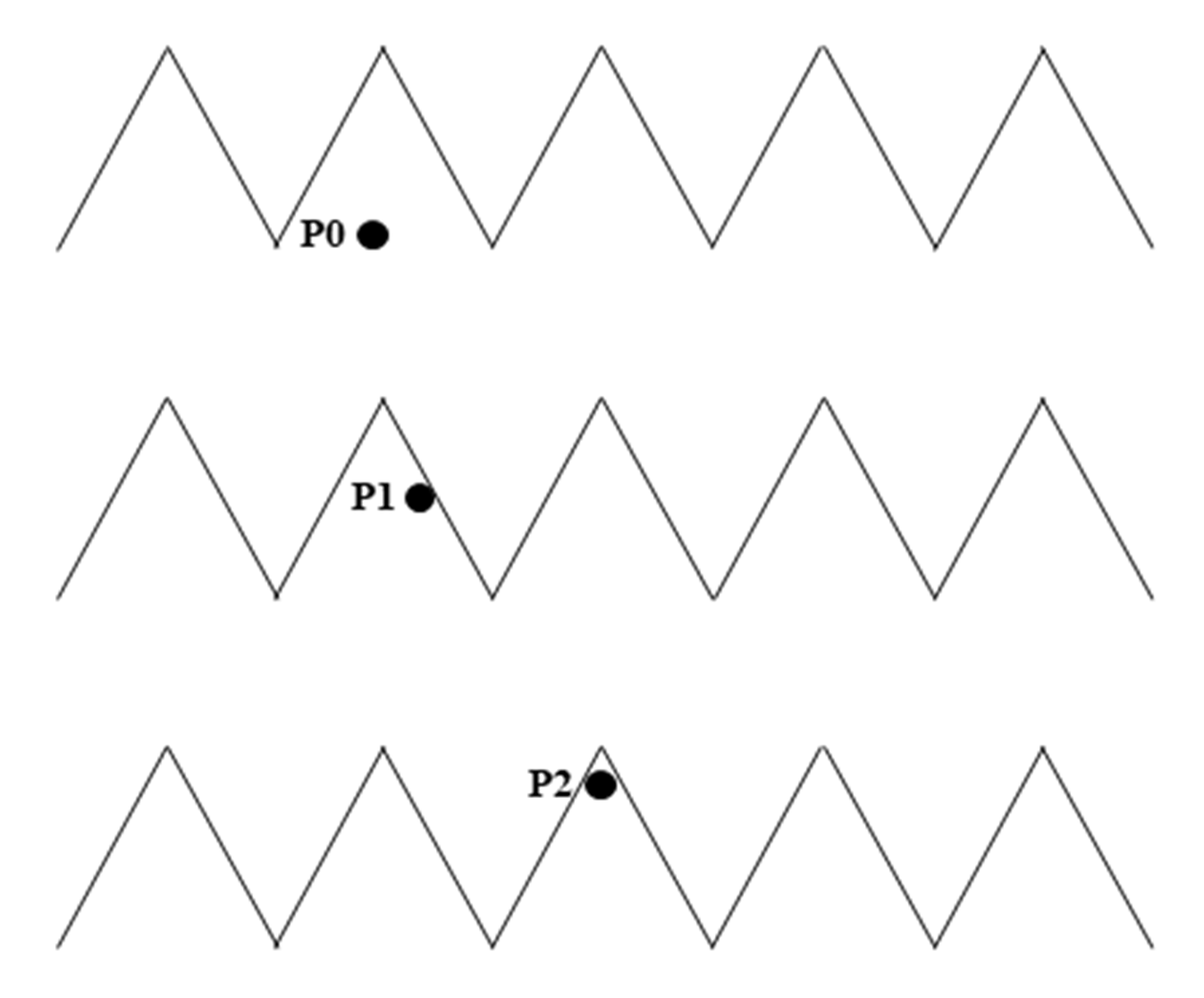

Supplement: Supplementary Figure S1 — Schematic diagram of different fenestration positions of LSA branch stent: P0: not affected by the metal structure of the main endograft; P1: affected by one metal structure of the main endograft; P2: Affected by two metal structures of the main endograft. [file Image_1.tif]

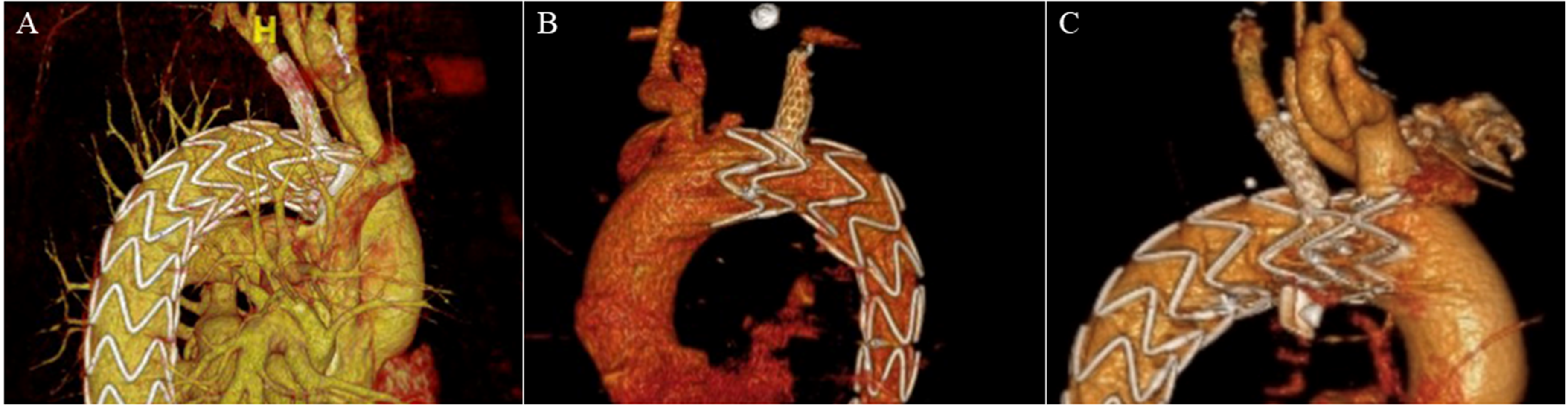

Supplement: Supplementary Figure S2 — Postoperative three-dimensional (3D) computed tomography angiography reconstruction image showed different fenestration positions of LSA branch stents. (A) LSA branch stent was not affected by metal structure (P0). (B) LSA branch stent was affected by one metal structure (P1). (C) LSA branch stent was affected by two metal structures (P2). [file Image_2.tif]
